# T-2 Toxin-induced Toxicity in Pregnant Mice and Rats

**DOI:** 10.3390/ijms9112146

**Published:** 2008-11-05

**Authors:** Kunio Doi, Noriaki Ishigami, Shinya Sehata

**Affiliations:** 1 Nippon Institute for Biological Science, 9-2221-1, Shin-Machi, Ome, Tokyo 198-0024, Japan; 2 Department of Veterinary Pathology, Graduate School of Agricultural and Life Sciences, The University of Tokyo, 1-1-1, Yayoi, Bunkyo, Tokyo 113-8657, Japan; 3 Fukui Safety Research Laboratories, Ono Pharmaceutical Co., Ltd., 5-10 Yamagishi, Mikuni-Cho, Sakai-Shi, Fukui 913-0032, Japan. E-Mail: ishigami@ono.co.jp; 4 Daiichi Sankyo Inc., 399 Thornall Street, Edison, NJ 08837, USA. E-Mail: ssehata@dsus.com

**Keywords:** T-2 Toxin, maternal toxicity, fetal toxicity, apoptosis, mouse, rat

## Abstract

T-2 toxin is a cytotoxic secondary fungal metabolite that belongs to the trichothecene mycotoxin family. This mycotoxin is a well known inhibitor of protein synthesis through its high binding affinity to peptidyl transferase, which is an integral part of the ribosomal 60s subunit, and it also inhibits the synthesis of DNA and RNA, probably secondary to the inhibition of protein synthesis. In addition, T-2 toxin is said to induce apoptosis in many types of cells bearing high proliferating activity. T-2 toxin readily passes the placenta and is distributed to embryo/fetal tissues, which include many component cells bearing high proliferating activity. This paper reviews the reported data related to T-2 toxin-induced maternal and fetal toxicities in pregnant mice and rats. The mechanisms of T-2 toxin-induced apoptosis in maternal and fetal tissues are also discussed in this paper.

## 1. Introduction

T-2 toxin is a cytotoxic secondary fungal metabolite that belongs to the trichothecene mycotoxin family. They are produced by various species of *Fusarium* (*F. sporotichioides, F. poae, F. equiseti, F. acuminatum*), which can infect corn, wheat, barley and rice crops in the field or during storage [1, 2]. T-2 toxin is conjectured to be a major factor in alimentary toxic aleukia in humans [3] and has been implicated in additional mycotoxicoses such as red mold disease in humans and animals [4] and bean-hull poisoning in horses [5].

T-2 toxin is a well-known inhibitor of protein synthesis through its high binding affinity to peptidyltransferase which is an integral part of the 60s ribosomal subunit [6–8]. Subsequent inhibition of the peptidyl transferase reaction can trigger a ribotoxic stress response that activates *c*-Jun *N*-terminal kinase (JNK)/p38 mitogen-activated protein kinases (MAPKs) [6]. T-2 toxin also inhibits the synthesis of DNA and RNA, probably secondary to inhibition of protein synthesis [8, 9]. Moreover, T-2 toxin interferes with the metabolism of membrane phospholipids and increases liver lipid peroxides [10, 11].

The toxic effects of T-2 toxin have been studied in experimental animals – poultry, cattle, sheep and pigs – all of which appear to be sensitive to this mycotoxin. Among farm animals, pigs are the most sensitive species [8], and ruminants are more resistant to the adverse effects of T-2 toxin due to microbial degradation within rumen microorganisms [12].

Oral, parenteral and cutaneous exposures to T-2 toxin induce lesions in hematopoietic, lymphoid and gastrointestinal tissues and suppress reproductive organ functions [13–16]. In addition, cardiomyopathy has been reported after topical application of T-2 toxin to rats [17], and signs somewhat similar to those in human alimentary toxic aleukia have been reported in rhesus monkeys and cats fed T-2 toxin [18]. The exact mechanism of T-2 toxin-induced lesions has remained unclear for many years. However, Quiroga *et al*. [19] have found that the thymocytes of T-2 toxin-inoculated mice undergo ultrastructural changes suggestive of apoptotic cell death. Thereafter, our research group showed in a series of experiments using adult mice and rats that T-2 toxin induces apoptotic cell death of lymphocytes in the thymus, splenic white pulp [20], and Peyer’s patches [21], hematopoietic cells in the bone marrow and splenic red pulp [22], intestinal crypt epithelial cells [23], and epidermal basal cells [24], suggesting that T-2 toxin can induce apoptosis in many types of cells bearing high proliferating activity. In addition, Shinozuka *et al*. [25] reported that T-2 toxin also induces apoptosis and fatty change in hepatocytes of mice following the increased expression of both oxidative stress-related, and apoptosis-related genes ( *c-fos* and *c-jun*). The elevated expression of oncogenes (*c-jun* and *c-fos*) as well as cytokines (TNF-alpha, TGF-beta1 and IL-1beta) is also reported in keratinocytes of rats topically applied with T-2 toxin [26–29].

T-2 toxin readily passes through the placenta and is distributed to embryo/fetal tissues which include many component cells bearing high proliferating activity [30]. This paper reviews the T-2 toxin-induced toxicity in pregnant mice and rats, although there are not so many reports of T-2 toxin-exposure to pregnant animals.

## 2. Maternal toxicity

Most of the reports of T-2 toxin-exposure to pregnant mice and rats are focused on embryo/fetal toxicity, with little reference to maternal toxicity [13, 30–40]. Sehata *et al*. [41, 42] examined the maternal toxicity in detail in pregnant rats exposed to a single oral dose of T-2 toxin (2 mg/kg) on day 13 of gestation. In their experiments, apoptosis was induced in lymphoid, hematopoietic and gastrointestinal tissues and liver as described in the above-mentioned reports in adult mice. It is said that the *c-fos* gene plays an important role in the early phase of T-2 toxin-induced apoptosis in the lymphoid and hematopoietic tissues probably through the synthesis of a certain apoptosis-related protein [43]. The elevation of *c-fos* expression requires the mobilization of [Ca^2+^]_i_ and partially involves a protein kinase C (PKC)-dependent pathway, and the mobilization of [Ca^2+^]_i_ activates calcium-dependent caspases, resulting in internucleosomal DNA fragmentation [44, 45]. T-2 toxin-induced apoptosis in hematopoietic and lymphoid tissues is considered to be independent of the Fas/Fas ligand pathway [43, 46] and the *p53*-related pathway [43].

Hemorrhage with apoptosis of cytotrophoblasts was observed in the placenta of a small percent of pregnant rats exposed to a single oral dose of T-2 toxin (2mg/kg) [41]. Placental hemorrhage is also reported in a small percent of pregnant mice exposed to a single oral dose of T-2 toxin (3 mg/kg) on either day 7, 8, 10, 11 or 12 of gestation [33, 36, 47]. The cause of such placental hemorrhage may be a result of the direct cytotoxic effect of T-2 toxin on the delicate vasculature in the labyrinth zone [41, 48], an effect of T-2 toxin on the clotting system, either by depressing clotting factors [49], or disturbing platelet function [50], or a combination of both [47]. In this regard, the prolongation of both prothrombin time and activated partial thromboplastin time and the decrease in the expression of blood coagulation-related genes (factors V, VII and X, kallikrein, and vitamin K epoxide reductase complex, subunit 1) are reported in adult mice exposed to a single oral dose of T-2 toxin (10 mg/kg) [25].

Rousseaux *et al*. [51] reported that no long-term reproductive and teratological effects of low dose dietary T-2 toxin (1.5 and 3.0 ppm) were found in the two-generation female reproduction and teratology study.

## 3. Fetal toxicity

As mentioned above, T-2 toxin readily passes through the placenta and is distributed to the fetal tissues [30], resulting in the induction of embryo/fetal death, fetal brain damage and fetal bone malformation [36]. In addition, thymic atrophy due to reduction in the number of CD44^low^ and CD45^low^ fetal liver prolymphocytic cells and prothymocytes and suppression of humoral immunity due to reduction in CD45R^+^B cell precursors have been reported in the mouse fetus from dams exposed to T-2 toxin from days 14–17 of gestation at dose levels (1.2 or 1.5 mg/kg) below that where other toxicities are observed [37, 38]. This indicates that the developing immune system may be a particular sensitive target of T-2 toxin exposure. Murine ontogenic development of thymocytes in the thymus originates from precursor cells in the fetal liver that seed the thynic rudiment on day 10–11 of gestation [52, 53], and the fetal thymus contains significantly greater proportions of immature proliferating thymocytes than are present in corresponding adult models [53]. The difference in susceptibility to T-2 toxin among lymphocyte subsets is also observed in adult mice [54], and the difference in the degree of lymphocyte apoptosis among lymphoid tissues reflects the difference in the lymphocyte population susceptible to T-2 toxin among lymphoid tissues [20, 54]. Conversely, Blakley *et al*. [35] reported that prenatal exposure to a single oral dose (0.75 or 1.5 mg/kg) of T-2 toxin on day 11 of gestation did not produce any impairment of humoral immunity and direct cytotoxic manifestations of T-2 toxin on antibody-producing cells were not observed, although the morphological and functional development of the murine immune system is said to be particularly sensitive to toxic insult during days 10–12 of gestation [55]. Blakley *et al*. [35] suggested that the embryolethal effects are a primary limiting factor which may preclude the expression of any immunoteratological manifestations associated with humoral immunity under natural field conditions.

It has been considered that T-2 toxin is primarily maternotoxic and embryolethal, and that defective development and induction of malformations are possibly secondary to maternal toxicity [13, 32, 36], although how the fetus is damaged by maternal toxicity is still unknown. On the other hand, Ishigami *et al*. [39] first reported that T-2 toxin (3 mg/kg) can induce apoptosis, especially in the central nervous and skeletal systems after oral administration to pregnant mice, indicating the direct cytotoxic effect of T-2 toxin on fetal tissues. They demonstrated that T-2 toxin-induced skeletal malformations and telencephalic lesions are greatly reduced by pretreatment with cyclohexamide, a protein synthesis inhibitor, as reported in thymocyte apoptosis in adult mice exposed to T-2 toxin (10 mg/kg) [43]. In addition, the number and region of apoptotic cells induced in the mouse fetus by T-2 toxin (3 mg/kg) vary according to the embryonic day. For example, apoptosis is observed in many neuronal progenitor cells and a small number of chondroblasts and chondrocytes on embryonic day 13.5 while it is detected in many cells in the thymus and renal subcapsular parenchyma on embryonic day 16.5 [40]. In rat fetuses from dams exposed to T-2 toxin (2 mg/kg) on day 13 of gestation, apoptosis is observed mainly in the central nervous system, liver (both hepatocytes and hematopoietic cells), gastrointestinal tract and cartilage primordium [41]. Apoptosis of hematopoietic cells in the fetal liver is considered to be similar to that in the spleen and bone marrow reported in adult mice treated with T-2 toxin (10 mg/kg) [22].

T-2 toxin is generally considered to induce apoptosis in actively proliferating cells in embryos and fetuses, probably through its radiomimetic effect. However, it should not be forgotten that a small number of apoptotic cells are also observed in some regions where proliferating cell nuclear antigen (PCNA)-positive cells are not detected [40], suggesting that T-2 toxin-induced apoptosis in the developing mouse fetus might also be affected by some other factors in addition to the proliferating activity of target cells. Similar findings are also reported in the intestinal crypt epithelial cells of adult mice exposed to T-2 toxin [23, 56].

Bone malformation such as incomplete ossification, absence of bones, wavy bones and fused bones that is one of the most frequently observed fetotoxicities of T-2 toxin [13, 32, 34, 36] is now considered to be related to T-2 toxin-induced apoptosis in the caudal half of the sclerotome around the notochord, and in the mesenchyme, chondroblasts and chondrocytes around cartilage primordium [40]. Judging from the results of *in vitro* studies, it is suggested that T-2 toxin (8 ng/mL) injures chondrocytes through superinduction of IL-1beta and IL-6 [57] and that apoptosis of chondrocytes can be induced by T-2 toxin (1–20 ng/mL) *via* the Bcl-2 and Bax proteins and the Bax/Bcl-2 ratio may play a critical role in governing the susceptibility to T-2 toxin-induced apoptosis in chondrocytes [58]. Prenatal exposure to T-2 toxin may also induce chondrocyte apoptosis in the fetus in the fetus through similar mechanisms, resulting in bone malformation.

As mentioned above, T-2 toxin passes through the placenta [30], and the blood brain barrier is not completely developed before embryonic day 18 in rats [59]. In addition, T-2 toxin and its metabolite, Ht-2, have a lipophilic nature and the fetal brain is rich in lipids. Therefore, T-2 toxin may be easily distributed to the fetal brain. Sehata *et al*. [60] have investigated the mechanisms of apoptosis induction in the fetal brain by the oral administration of T-2 toxin (2 mg/kg) to pregnant rats on day 13 of gestation. The number of apoptotic neuronal progenitor cells in the telencephalon increased at 1 hr and peaked at 12 hr. Based on the results of DNA microarray analysis and real time PCR done on the fetal brain at 6, 12 and 24 hr, they concluded that the T-2 toxin-induced toxicity in the fetal brain is due to oxidative stress, and that MAPK pathway (especially MEKK1 and *c-jun*) is involved in T-2 toxin-induced apoptosis in the fetal brain. In addition, the increase in caspase-2 gene expression with no changes in caspase-9 and Bax-alpha gene expression was also detected, suggesting an involvement of caspase-2 activation in T-2 toxin-induced apoptosis in the fetal brain. Acivation of caspase-2 is induced by reactive oxygen species, and casepase-2 is said to play a crucial role in the control of apoptosis [61, 62]. It is also said that activation of caspase-2 is essential to T-2 toxin-induced apoptosis and that apoptotic signals are mainly transmitted *via* caspase-8 and caspase-3 rather than mitochondrial pathway [63]. On the other hand, apoptosis induction in the fetal brain by T-2 toxin seems to be independent of the p53-related pathway which is the most important pathway in DNA-damaging agent-induced apoptosis of neuronal progenitor cells in the developing brain [64–68].

## 4. Relationship between maternal and fetal toxicities

**Figure 1.**
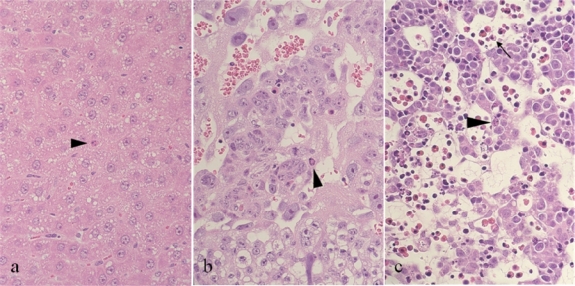
Histopathology of maternal liver (a), placenta (b) and fetal liver (c) obtained from pregnant rats at 6 hr after treatment with T-2 toxin (2 mg/kg) on day 13 of gestation. An arrowhead indicates apoptotic hepatocyte (a and c) and cytotrophoblast (b), and an arrow indicates apoptotic hematopoietic cell (c). Apoptotic cells show pyknotic or karyorrhectic nucleus and condensed eosinophilic cytoplasm. Hematoxylin and eosin stain, × 100 (original magnification).

Sehata *et al*. [41] reported that prenatal exposure to T-2 toxin (2 mg/kg) induces apoptosis in maternal tissues, placenta and fetal tissues. For example, apoptotic cells bearing pyknotic or karyorrhectic nucleus and condensed eoshinophilic cytoplasm are observed in maternal liver (hepatocytes), placenta (cytotrophoblasts) and fetal liver (hepatocytes and hematopoietic cells) (Figure 1). Based on the results of microarray analysis at 23 hr after T-2 toxin (2 mg/kg) exposure on day 13 of gestation, they suggested that T-2 toxin induces oxidative stress in these tissues following the changes in metabolism–related gene expression and that these changes may alter the intracellular environments resulting in the induction of apoptosis [69]. They further investigated the mechanisms of apoptosis in the maternal liver, placenta and fetal liver at earlier time points [70].

**Figure 2.**
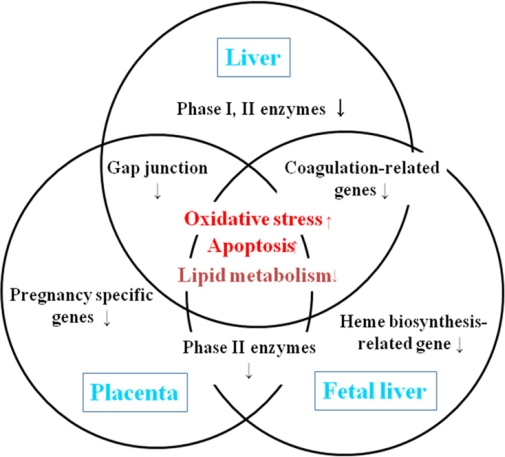
Summary of DNA microarray analysis done on maternal liver, placenta and fetal liver obtained from pregnant rats treated with T-2 toxin on day 13 of gestation.

The apoptotic index peaked at 6 hr and at 12 hr in the maternal liver and placenta, respectively, and it decreased thereafter. In the fetal liver, it reached a plateau at 12 hr. Microarray analysis done on these tissues at 3, 6 or 12 (peak time point of apoptosis), and 24 hr showed changes in the expression of many genes. Increased expression of oxidative stress- and apoptosis-related genes was commonly detected in these three tissues at the peak time point of apoptosis, and decreased expression of lipid metabolism- and drug-metabolizing enzyme-related genes was also commonly detected in these three tissues (Figure 2). Therefore, the mechanism of T-2 toxin-induced toxicity in pregnant rats is considered to be due to oxidative stress followed by the activation of MAPK pathway, finally inducing apoptosis, as reported in the fetal brain [60].

Oxidative stress is certainly involved in the toxicities of trichothecene mycotoxins including T-2 toxin [71], MAPKs may play integral roles in the diverse toxic manifestations of trichotecenes [72], and ribosome binding or protein synthesis inhibition may play roles in MAPK activation and apoptosis induction by trichothecenes [6, 72]. Oxidative stress causes lipid peroxidation and induces mitochondrial dysfunction which causes fatty acid *β*-oxidation and induces fatty liver [73]. In addition, T-2 toxin enhances lipid peroxidation [10, 74]. Therefore, the disturbance of lipid metabolism caused by oxidative stress may occur in the maternal liver, placenta and fetal liver by T-2 toxin [70].

Increased expression of Bax-α as well as p53 was detected in maternal and fetal livers [70]. Bax, one of the p53’s target genes, is a member of the bcl-2 family and induces apoptosis, and apoptosis induction in HL60 cells by T-2 toxin involves activation of caspase-3 and –9 through the release of cytochrome c from mitochondria in the cytosol [76], suggesting the involvement of p53-related mitochondrial pathway in the T-2 toxin-induced apoptosis at least in the maternal and fetal livers. On the other hand, differing from the above-mentioned case of T-2 toxin-induced apoptosis in the fetal brain [60], increased expression of casepase-2 gene was not detected in the maternal liver, placenta and fetal liver. In addition, unlike DNA-damaging agent-induced placental apoptosis [75], p53 was not involved in T-2 toxin-induced placental apoptosis [70].

The number of metabolism-related genes in the placenta is smaller than those in the maternal liver and fetal liver, suggesting that the placenta might play a less role in T-2 toxin metabolism. In addition, the differences in the expression of cytochrome P-450 genes between the maternal liver and fetal liver might reflect the difference in the basic expression of cytochrome P-450 genes between the maternal liver and fetal liver.

## 5. Conclusions

**Figure 3.**
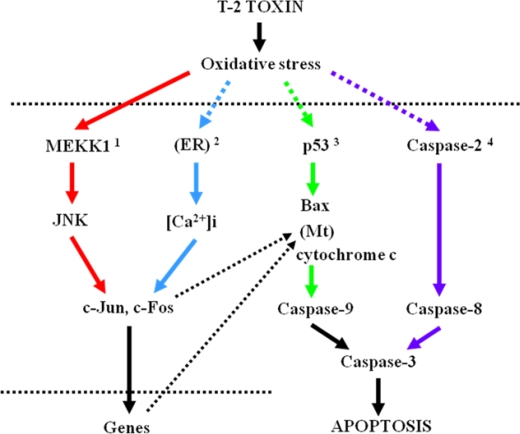
Hypothesis of mechanisms involved in T-2 toxin-induced apoptosis in maternal and fetal tissues. ER: endoplasmic reticulum; Mt: mitochondrion. ^1^ Main pathway, ^2^ Additive pathway in hematopoietic and lymphoid tissues, ^3^Additive pathway in maternal and fetal liver, ^4^Additive pathway in fetal brain.

T-2 toxin readily passes the placenta and directly affects the fetus, resulting in the induction of apoptotic cell death mainly in the fetal lymphoid, central nervous and skeletal systems and liver. In dams, T-2 toxin induces apoptotic cell death in the placenta in addition to the tissues which are reported to be sensitive to T-2 toxin in adult mice and rats. The mechanisms of T-2 toxin-induced maternal and fetal toxicities are due to oxidative stress, followed by activation of the MAPK pathway, finally inducing apoptotic cell death (Figure 3). However, there are some differences in additive pathways involved in T-2 toxin-induced apoptosis among tissues affected (Figure 3).
